# Looking Back Over 40 Years: A Personal Journey and Our Specialty

**DOI:** 10.1055/s-0046-1827060

**Published:** 2026-08-12

**Authors:** Mukund Thatte

**Affiliations:** 1 Head of the Department of Plastic Surgery, Bombay Hospital & Medical Research Centre, Mumbai

This is indeed a very interesting request of the honorary editor. What do I reflect on? Our association, our specialty, or our future. Perhaps a bit of everything.

## My Journey

I qualified in 1986 from the Lokmanya Tilak Municipal General Hospital and Medical College, also known as “Sion Hospital” in Mumbai. I immediately joined the Association of Plastic surgeons of India (APSI)—late Dr. Mathangi Ramakrishnan was treasurer and Dr. K. Sekhar was honorary secretary. Under the freshly promulgated constitution of 1986, they immediately gave me full membership once I qualified.

I was fortunate to attend IPRAS 1987 as a young member at reduced cost and see for myself the living legends of plastic surgery, such as Millard, Tessier, Ortiz Monasterio, Ian Jackson, and many others. I also saw the transformation of the APSICON from a small, intimate meeting to the vast meeting it has become today.

## Association of Plastic Surgeons of India (APSI) Participation

After being the organizing secretary of APSICON 1994, I was elected to the executive council. I served from 1995 to 1997. The guidelines for the PEET prize were formulated by me and Dr. K. Sridhar at the request of President Dr. Ramakrishnan Nair in 1995.

In 2002, I got elected as editor for the *Indian Journal of Plastic Surgery* . I decided to take up the editorship of our journal because I felt deeply that, at the end of the day, an APSICON is only a memory. The journal endures as a legacy of the APSI. Initially, we got three copies typed double-spaced with three copies of photos by post, sent to reviewers by post, and later correspondence with authors by post. In 2003, this was an anachronism. I was able to move it online including manuscript management. The journal transformed itself rapidly; however, we never compromised on free full-text access to readers. We are one of the few journals that do not charge authors or readers for access. This has resulted in us getting searched very quickly on PubMed, as free full-text papers get preference. I am very glad that subsequent editors have proudly upheld this tradition.

I was fortunate to be chosen by our members as their president in APSICON 2008 at Varanasi. We did the APSICON 2010 in Goa. We hoped to combine fun, friendship, social interaction, and science. Although I say it myself, we were successful. However, I remember APSICON 2010 because we finally sorted the running dispute between the APSI trust and the 9th IPRAS fund; this had been a sore on our body politic from 1995 till 2010. Every APSICON GBM ended in acrimony over this issue. With the help of the then executive trustee Dr. K. Sridhar, we managed to convince both sides to merge once and for all and ended the very unfortunate dispute. I personally think this was the landmark historical moment in my tenure as president.

## Evolution of Skills in the Country

The APSICON today is unrecognizable sometimes due to the new topics, new technology, and focus on aesthetic surgery. We grew up doing clefts, burn contractures, tissue defects, and hand work. Aesthetic surgery was a distant entity jealously guarded by a few. It was similar to microsurgery. However, denial of training to juniors never stops them from learning, and very soon there was an entire group of people who had obtained training in aesthetic surgery and microsurgery. They broke the mystique and made these things mainstream. I feel I helped in that regard with free tissue transfer along with colleagues, such as Drs. S.M. Kumta, N.J. Mokal, and A.S. Baliarsing in the city of Mumbai. Others did the same elsewhere in India.

In the 1990s, our focus shifted to brachial plexus injury, a field that was largely neglected then. Some of us entered as self-taught specialists and, through sustained clinical practice and gradual refinement of techniques, contributed to its evolution into a recognized mainstream discipline.

APSI evolved with these changes. In many respects, it expanded in scope and strengthened its role, progressively incorporating and catering to emerging modalities within the specialty.

## Legal Issues

Plastic surgery has always simplified and solved problems. Over time, regional specialties lay claim to these procedures, creating unease and heartburn among plastic surgeons. Hypospadias is a classic example of our specialty losing a procedure over time. Our current dispute with dental colleagues is a case in point. The courts will, of course, give judgment over our plea as well as the government.

What is needed is for the government to first recognize APSI, ASI, and such national bodies as legitimate, official, and valid stakeholders in these matters, much like the royal colleges are in the United Kingdom. Till that is done, we will continue to be supplicants appealing to the politicians and bureaucrats who frankly do not understand the minutiae, and, I believe, do not really care.

Although this sounds clichéd, I have observed in the last 40 years that ultimately your results speak for themselves. I believe if we deliver consistent good results with minimal complications and adopt newer safe techniques vigorously, no one can take away our work.

I believe plastic surgery will keep evolving, but the basic thought process will still underpin the newer modalities.

## Notes

**Note:** This article has been corrected by publishing a correction notice with doi: 10.1055/s-0046-1829879.

