# Erratum: Looking Back Over 40 Years: A Personal Journey and Our Specialty

**DOI:** 10.1055/s-0046-1829879

**Published:** 2026-10-26

**Authors:** Mukund Thatte

**Affiliations:** 1 Head of the Department of Plastic Surgery, Bombay Hospital & Medical Research Centre, Mumbai

It has been brought to the publisher's attention that the name the author's name was published incorrectly in the above article, published in the *Indian Journal of Plastic Surgery* , Volume 59, Issue 3 on August 12, 2026 (doi: 10.1055/s-0046-1827060 ).

The author's name has been corrected in the online version of the article and is correctly stated above.

